# Sidewall Aneurysm Geometry as a Predictor of Rupture Risk Due to Associated Abnormal Hemodynamics

**DOI:** 10.3389/fneur.2019.00841

**Published:** 2019-08-14

**Authors:** Hailin Wan, Liang Ge, Lei Huang, Yeqing Jiang, Xiaochang Leng, Xiaoyuan Feng, Jianping Xiang, Xiaolong Zhang

**Affiliations:** ^1^Department of Radiology, Huashan Hospital, Fudan University, Shanghai, China; ^2^ArteryFlow Technology Co., Ltd., Hangzhou, China

**Keywords:** intracranial aneurysms, hemodynamics, geometry, computational fluid dynamics, rupture

## Abstract

**Background:** Hemodynamics play an important role in intracranial aneurysm (IA) initiation, growth, and rupture. Yet there remains no definitive quantitative analysis between abnormal hemodynamics and geometrical risk of IA development.

**Objective:** The present study aims to investigate whether abnormal hemodynamics in IA sacs can be predicted by surrogate geometric markers.

**Methods:** Computational fluid dynamics (CFD) simulations were performed on paraclinoid aneurysms derived from digital subtraction angiography (DSA) of 104 IAs in 104 patients. Four basic IA geometric parameters including maximum height, perpendicular height, maximum width, and neck diameter were measured. Abnormal hemodynamics were defined and quantified as the surface area exposed to low wall shear stress (WSS) and high oscillatory shear index (OSI) using objectively-defined thresholds. Relationships between abnormal hemodynamics and specific geometric parameters were analyzed via multiple linear regression.

**Results:** Adjusting for age, sex, and other clinical characteristics, multiple linear regression revealed a significant relationship (*p* < 0.001) between abnormal hemodynamics and both maximum width (β ≈ 1.2) and neck diameter (β ≈ −0.4), but not maximum height or perpendicular height. These findings were shown to be independent of the choice of abnormal hemodynamic indicators and threshold levels.

**Conclusions:** Maximum width and neck diameter of IA sacs are robust surrogates of exposure to abnormal hemodynamics. Risk of rupture may be increased with wider aneurysms with narrower necks for paraclinoid aneurysms.

## Introduction

Hemodynamics play an important role in intracranial aneurysm (IA) initiation, growth, and rupture (1–5). Recent studies using image-based computational fluid dynamics (CFD) modeling and statistical analyses have identified connections between the hemodynamic properties of intracranial aneurysms and the likelihood of their growth and rupture (2, 3), albeit with conflicting findings regarding wall shear stress (WSS) (6, 7). Currently, it is widely accepted that abnormal hemodynamics, especially low WSS, and high oscillatory shear index (OSI), promote IA rupture (3, 8). In addition, morphologic metrics have been explored to evaluate the aneurysmal rupture risk (9–11). However, definitive quantitative analysis between abnormal hemodynamics and geometrical parameters for IA growth and rupture remain unknown. This study aims to investigate whether surrogate geometric markers are predictive of abnormal hemodynamics in unruptured paraclinoid IAs.

## Methods

### Patient Selection

This retrospective study was approved by the ethics committee of Huashan Hospital affiliated to Fudan University, which waived the requirement for the informed patient consent. Three-dimensional angiographic images of patients with IAs diagnosed or treated at our center from April 2015 to June 2018 were reviewed. All collected images were examined for suitability for inclusion in the study. Only cases in which the 3-dimensional images were of sufficient quality for accurate segmentation and reconstruction were included. Unilateral multiple IAs, ruptured IAs, and IAs treated with endovascular embolization or surgical clipping were excluded. One hundred four paraclinoid aneurysms in 104 patients met such criteria and were consequently included in this study. All aneurysm geometries were measured from digital subtraction angiography (DSA).

### Morphological Parameter Calculations

The most common clinically used geometrical parameters—maximum height, perpendicular height, neck diameter, and maximum width—were measured via 3-dimensional angiographic images. Size, aspect, height-width, and height-neck ratios were not included, since they are calculated metrics derived from the above four basic geometrical parameters. In addition, the parent artery parameters, such as parent artery diameter, inflow and aneurysm angle, were not evaluated in this study, because the single specific paraclinoid location was chosen to eliminate parent artery variables.

### CFD Modeling

Each 3D aneurysm geometric model was imported and meshed using Star-CCM+ (CD Adapco, Melville, NY, USA) to create ~1 million polyhedral elements with maximum mesh size of 0.1 mm and four layers of wall prism elements for accurate boundary layer resolution. Incompressible Navier-Stokes equations were solved numerically under pulsatile flow conditions using Star-CCM+. Published mean flow rate of 4.6 ml/s was used as an inlet boundary condition since all the computational models originated from the internal carotid artery (ICA) (12). Pulsatile velocity waveform was obtained from Transcranial Doppler (TCD) ultrasound measurement on a normal subject, with the magnitude scaled to the desired mean flow rate (3). Traction-free boundary conditions were implemented at each outlet and the mass flow rate through each outlet artery was proportional to the cube of its diameter, based on the principle of optimal work (13). Blood was modeled as a Newtonian fluid with a density of 1,056 kg/m^3^ and a viscosity of 0.0035 N s/m^2^, while the Newtonian effect is negligible in cerebral main vessels. A rigid-wall and no-slip boundary condition was implemented for each vessel wall. Three pulsatile cycles were simulated to ensure that numeric stability was achieved, and the simulation results from the last cycle was taken as the output.

### Hemodynamic Parameter Calculations

While it is widely accepted that low WSS and high OSI promote IA rupture, there remains no definitive quantitative investigation between abnormal hemodynamics and the geometry of IA sacs. Applying the protocol of Lee et al. (14) 104 IA surfaces were aggregated to identify the threshold values of OSI and WSS. The sensitivity of the findings were tested for each threshold value by choosing 80 and 90% cumulative surface exposure. For each model, abnormal hemodynamics were quantified as surface area (SA) exposed to WSS below 80 or 90%, or OSI above 80 or 90%.

### Statistical Analysis

Continuous variables were expressed as the mean ± standard deviation (SD), whereas categorical variables were expressed as frequency and percentage. Adjusting for age, sex and other vascular risks, multiple stepwise linear regression was used to quantify the relationship between SA exposure to abnormal hemodynamics and maximum height, perpendicular height, maximum width, and neck diameter as independent predictors. Regression quality was analyzed using the Pearson correlation coefficient, adjusted by the number of independent variables ($R_{\text{adj}}^{2}$). The standardized regression coefficients (β) determined the relative magnitude of the geometric predictors. To test the sensitivity of findings for the selected abnormal hemodynamic indicators, the four permutations of hemodynamic parameters and threshold criterion (hereafter designated as WSS80, WSS90, OSI80, and OSI90) were analyzed separately. The Pearson correlation coefficient was used to assess the relationship between the obtained geometric and hemodynamic parameters. Two-sided *P* values of <0.05 were considered to indicate statistical significance. All analyses were conducted using IBM SPSS statistics for Windows, Version 19.0 (IBM Corp, Armonk, NY, USA).

## Results

The cohort consisted of 104 paraclinoid IAs from 104 patients with an average IA size of 6.06 ± 3.78 mm (range 1.68–20.02 mm). The mean age was 56.82 ± 9.99 years, ranging from 21 to 80 years. There were 86 female patients (82.7%), 36 with hypertension (34.6%), 10 smokers (9.6%), 9 with diabetes mellitus (8.7%) and 3 heavy drinkers (2.9%). In this study, we defined aneurysms larger than 7 mm as large aneurysms, accounting for 27.9% (29/104). Morphologic and hemodynamic parameters are presented in Table 1.

**Table 1:** Geometric and hemodynamic parameters.

*n* = 104	Minimum	Maximum	Mean	Std. deviation
Maximum height (mm)	1.68	20.02	6.06	3.78
Maximum width (mm)	2.71	22.24	6.82	3.75
Neck diameter (mm)	2.25	13.03	5.62	2.32
Perpendicular height (mm)	1.48	18.50	5.54	3.54
WSS (Pa)	0.71	18.60	5.34	3.62
OSI	0.0007	0.059	0.012	0.013

### WSS and OSI Distribution and Their Correlations With Morphology

**Figure 1:**
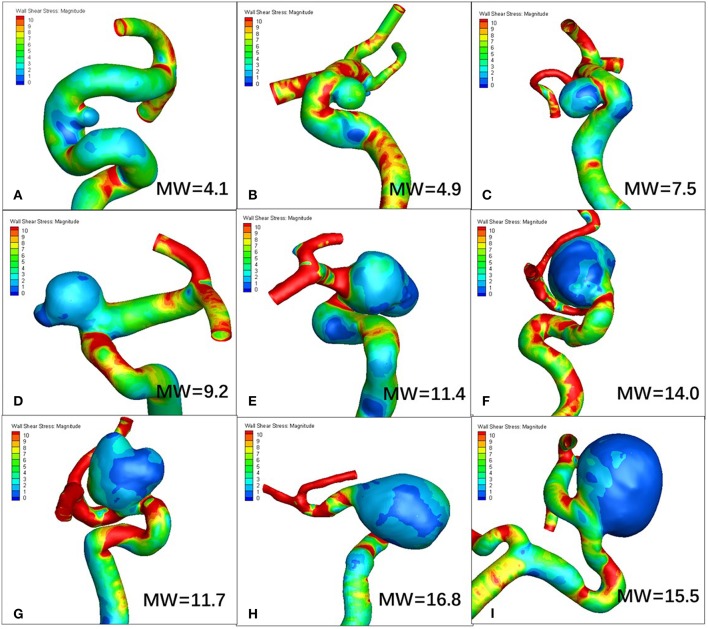
As maximum width (MW) increases, wall shear stress (WSS) tends to decrease. **(A–I)** are representative cases.

**Figure 2:**
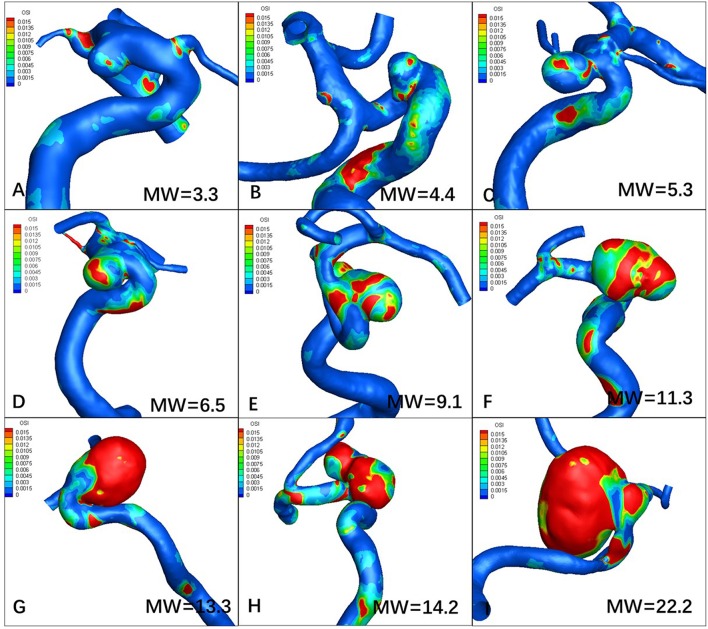
Increasing maximum width (MW) tends to increase oscillatory index (OSI). **(A–I)** are representative cases.

**Figure 3:**
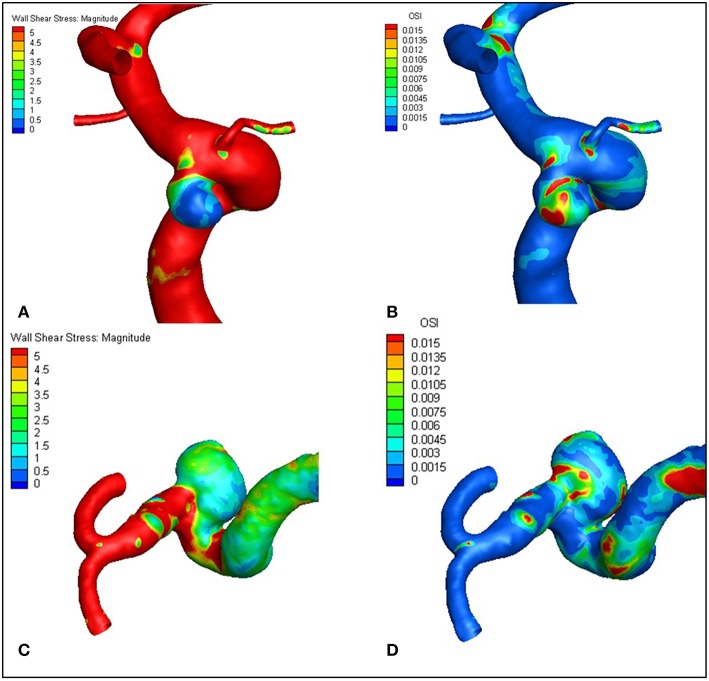
The narrow-necked aneurysm (top row) shows lower WSS and higher OSI, compared with a wide-necked aneurysm (bottom row). **(A–D)** are representative cases.

Figures 1, 2 visualize WSS and OSI distribution in different IA maximum width. We observed that as the IA maximum width increased, WSS tended to decrease and OSI tended to increase. Figure 3 demonstrated that the narrow-necked aneurysm (top row) had lower WSS and higher OSI, compared with a wide-necked aneurysm (bottom row). Maximum width was significantly associated with WSS and OSI (*R* = −0.56, *P* < 0.001; *R* = 0.51, *P* < 0.001, respectively), while neck diameter also significantly correlated with WSS and OSI (*R* = −0.53, *p* < 0.001; *R* = 0.48, *p* < 0.001, respectively).

In addition, through correlation analysis between WSS and OSI, we found that low WSS and high OSI were highly correlated, with Pearson R values of 0.88 (WSS80 vs. OSI80) and 0.87 (WSS90 vs. OSI90), which are both highly statistically significant (*P* < 0.001).

### Relationship Between IA Geometry and Abnormal Hemodynamics

A Multiple linear regression model quality is presented in Table 2. The degree of fit ($R_{\text{adj}}^{2}$) in the four models were very high (all *P* < 0.001). As summarized in Table 3, multiple regression analysis revealed a significant positive relationship between exposure to abnormal hemodynamics and maximum width, and a significant inverse relationship with neck diameter, but not maximum height and perpendicular height, irrespective of any hemodynamic parameter or threshold criterion.

**Table 2:** Multiple linear regression model quality.

	**Threshold value**	$R_{\text{adj}}^{2}$	***P* value**
WSS80	2.32 Pa	0.85	<0.001
WSS90	2.02 Pa	0.83	<0.001
OSI80	0.019	0.68	<0.001
OSI90	0.033	0.63	<0.001

**Table 3:** Multiple linear regressions of Surface area (SA) exposure to abnormal hemodynamics with maximum width, neck diameter, maximum height, and perpendicular height.

Standardized coefficients								
	β_width_	*P* value	β_neck_	*P* value	β_MH_	*P* value	β_PH_	*P* value
SA_WSS80_	1.26	<0.001	−0.38	<0.001	−0.057	NS (0.69)	0.003	NS (0.98)
SA_WSS90_	1.27	<0.001	−0.42	<0.001	−0.104	NS (0.50)	0.033	NS (0.80)
SA_OSI80_	1.21	<0.001	−0.45	0.001	0.093	NS (0.66)	0.24	NS (0.19)
SA_OSI90_	1.22	<0.001	−0.51	<0.001	0.16	NS (0.47)	0.31	NS (0.11)

*NS, not significant*.

## Discussion

Adjusting for age, sex, and other vascular risk factors, this study quantified the relationship between surface area (SA) exposed to abnormal hemodynamics and basic geometrical parameters of aneurysmal sacs. Our study has determined that SA exposure to abnormal hemodynamics can be predicted by a relatively simple formula:

(1) $$\begin{array}{r} \text{SA }\infty \;\beta 1\;\times \text{ maximum width}-\beta 2\;\times \text{ neck diameter} \end{array}$$

where β is the contribution of each selected independent variable to abnormal hemodynamics. That is, IAs with large maximum width are more susceptible to abnormal hemodynamics, which may be offset by an increase in neck diameter. To our knowledge, this study is one of the first to quantitatively investigate the relationship between abnormal hemodynamics and IA geometry.

Image-based CFD models have suggested correlations between abnormal hemodynamics and intracranial aneurysm growth and rupture, albeit with conflicting findings regarding WSS (2, 6, 15, 16). Recent studies have shown that low WSS and high OSI may facilitate IA growth and rupture (2, 3, 17). Meng et al. postulated that low WSS and high OSI trigger an inflammatory cell mediated pathway, which could be associated with the growth and rupture of large, atherosclerotic aneurysm phenotypes (6, 18). In addition, Varble et al. found that ICA aneurysms had lower WSS areas compared with other non-ICA aneurysms (19). Furthermore, Can et al. found that the location of the aneurysm at the bifurcation or sidewall may influence the correlation of such hemodynamics (5). Therefore, this location-specific study, investigating paraclinoid aneurysms, defined abnormal hemodynamics as low WSS and high OSI.

Our study demonstrated a significant positive relationship between surface area exposure to abnormal hemodynamics and maximum width, and a significant inverse correlation with aneurysmal neck diameter. Qiu et al. found that aneurysms with varying neck widths have different hemodynamics (20). Our results are consistent with their hypothesis that when the aneurysm width is greater than the neck width, the inflow and outflow are relatively lower and the impact on blood velocity flow is also reduced, leading to lower WSS. Furthermore, the bottleneck factor, simultaneously using aneurysmal width and neck diameter, was positvely correlated with the risk of rupture in IAs (10). Therefore, the combination of maximum width and neck diameter may be useful predictors of IA rupture.

Several limitations should be noted in this study. First, our patient population derived from a single center and only paraclinoid aneurysms were investigated, and thus selection bias may exist and the conclusion from this study may not be generalized. Second, due to clinical practicality, only four basic geometrical parameters were selected as independent variables, while other computative metrics and complicated three-dimensional parameters, such as undulation index and non-sphericity index, were excluded. Third, parent artery properties such as vessel diameter, curvature, inflow angle, and aneurysm angle were not evaluated. In this study, we focused only on paraclinoid IAs to eliminate parent artery variables. Fourth, we adopted several commonly used assumptions to make CFD tractable. Because of a lack of patient-specific information, we assumed a generic inlet waveform and a constant location-based inlet flow rate. The inlet velocities were scaled according to the inlet diameter. We further assumed that blood functioned as a Newtonian fluid and IAs have rigid walls. Fifth, as this study focused on pretreatment CFD models, further research can be undertaken on aneurysmal progression and follow up.

## Conclusion

In paraclinoid aneurysms, maximum width and neck diameter of IA sacs are robust surrogate markers of exposure to abnormal hemodynamics, indicating that IAs with large maximum width and small neck diameter may be prone to rupture. Further study can be extrapolated and extended to other specific IA sites.

## Data Availability

The raw data supporting the conclusions of this manuscript will be made available by the authors, without undue reservation, to any qualified researcher.

## Ethics Statement

Approval for the study was obtained from the review board of Huashan Hospital affiliated to Fudan University.

## Author Contributions

XF, XZ, and JX designed this study. LG, LH, and YJ analyzed CFD results. HW, JX, and XL performed the CFD study. HW and LG wrote the manuscript.

### Conflict of Interest Statement

JX and XL are employed by ArteryFlow Technology Co. The remaining authors declare that the research was conducted in the absence of any commercial or financial relationships that could be construed as a potential conflict of interest.
